# *In-vitro* Chemopreventive Potential of a Chromone from *Bomarea setacea (*ALSTROEMERIACEAE) against Colorectal Cancer 

**DOI:** 10.22037/ijpr.2020.113745.14466

**Published:** 2021

**Authors:** Angie Herrera-R, Gustavo Moreno, Pedronel Araque, Isabel Vásquez, Elizabeth Naranjo, Fernando Alzate, Wilson Cardona-G

**Affiliations:** a *Química de Plantas Colombianas, Institute of Chemistry, Faculty of Exact and Natural Sciences, University of Antioquia, UdeA, Calle 70 No. 52–21, A.A 1226, Medellín, Colombia. *; b *Grupo de Investigación e Innovación en Formulaciones Químicas, Escuela de ciencias de la vida, EIA University, km 2 + 200 Vía José María Córdova airport, Postal Code 055428, Envigado, Colombia. *; c *Grupo de Estudios Botánicos, Institute of Biology, Faculty of Exact and Natural Sciences, University of Antioquia UdeA, A.A. 1226 Medellín, Colombia.*

**Keywords:** Antiproliferative, Apoptosis, Bomarea setacea, Chromone, Colorectal cancer

## Abstract

Chemoprevention with natural products may provide important alternatives in the search for new drugs to treat cancer. Thus, the ethanol extract of *Bomarea setacea *and its secondary metabolite (chromone) were evaluated *in-vitro* in SW480 and SW620 human adenocarcinoma colon cells to identify a possible effect on cell growth, antiproliferative and/or proapoptotic activity. The ethanol extract did not show growth inhibition of these cell lines 48 h after treatment; besides, it required higher concentration and time to have an antiproliferative effect. On the other hand, although the chromone was not as active as the reference drug (5-FU), it displayed a greater selectivity, being 156-fold more selective against SW480 cells (SI => 100) and 255-fold against SW620 cells (SI => 86,9). Additionally, the chromone caused an important arrest in G2/M (44.18%) with an important accumulation in subG0/G1 phase in SW620 cells, inducing loss in mitochondrial membrane potential and damage in the cell membrane of both cell lines, with activation of caspase 3, suggesting an apoptotic process independent of ROS production and p53 activation.

## Introduction

Despite colorectal cancer (CRC) can be highly preventable through changes in lifestyle (1), this is still a leading cancer-related cause of death worldwide, being the second most common, accounting for 935,173 deaths in 2020, only preceded by lung cancer (2). Many risk factors contribute to colorectal cancer development, including smoking, alcohol consumption, physical inactivity, diets high in fat and red meats, inadequate intake of dietary fiber, vegetables and fruits, and obesity. 

Current treatments for CRC such as FOLFIRI (folic acid/5 FU/irinotecan), FOLFOX (5-FU/leucovorin/oxaliplatin) and FOLFOXIRI (leucovorin/5-FU/oxaliplatin/irinotecan) include combinations of chemotherapeutic agents which are composed of 5 fluorouracil as the backbone of the treatment. These therapeutic schemes are effective; however, they cause undesirable neurological and gastrointestinal side effects, which often result in dose limitations or cessation of the anticancer therapy (3-5). Due to the increase in the statistics and the toxicity associated with conventional chemotherapy, extensive research is ongoing to develop new pharmaceutical agents with chemopreventive potential against colorectal cancer. Among the studies carried out, the use of natural products may provide important alternatives in the search for new drugs to treat these diseases. On this matter, *the Bomarea *genus, which has 120-130 species distributed mostly in the neotropics, has exhibited some biological activities. *Bomarea* *setacea* (*B. setacea*) species belongs to the Alstroemeriaceae family, is a scandent herb reaching up to 2.5 m in length. The leaves are simple, alternate and typically develop epidermal projections beneath. The inflorescences are umbels with 5-35 flowers, externally red and internally yellow. This species has a wide geographic distribution in the Andes, including Colombia, Ecuador and Venezuela, at an elevation between 1900 and 4000 meters (6). Several species of *Bomarea* have been traditionally used locally as contraceptives by indigenous Amazonian communities. Little ethnobotanical information is known about this genus and less about its biological potentialities. It has been reported the antiprotozoal activity of the ethanol extract of the leaves of *Bomarea setacea* against promastigote form of three *Leishmania* species (*L. amazonensis *L., *L. braziliensis *Viana* and L. donovani*) (7). Previous studies have shown antioxidant activity of ethanol extracts from 11 species of the genus *Bomarea *through *in-vitro *methods (DPPH and TBARS) (8); besides, Gamal-Eldeen *et al.* (9). reported anticancer activity against hepatocellular carcinoma, immunoproliferative activity via induction of T-lymphocytes and macrophage proliferation, anti-inflammatory activity as indicated by nitric oxide inhibition and antioxidant activity against DPPH. On the other hand, Suárez *et al.* (10) reported the inhibitory effect of extract of *B. cornigera* in the development of solid tumors in mice. 

Based on the above*,* this study was aimed to isolate, characterize and evaluate the biological activity of the secondary metabolite (2-henicosyl-5,7-dihydroxy-4H-chromen-4-one, hereafter termed the chromone) from *Bomarea setacea*, using an *in-vitro* model of colorectal cancer, to identify possible therapeutic approaches for the treatment of colorectal cancer. 

## Experimental

*Plant materials*

*Bomarea setacea *was collected in a cloud Andean forest at an elevation of 2500 meters, close to the city of Medellin in the northwest of Colombia. Framework contract No. 234, RGE 289. Voucher specimen is kept at the University of Antioquia Herbarium (HUA) under number 182477. 

*Extraction and purification*

The material was dried in an oven at 35 °C for 48 h. Powdered leaves (168.2 g) of *Bomarea setacea *were extracted with ethanol in a percolator at room temperature and concentrated in a vacuum to give the corresponding extract (17 g, 10.1%). Then, this extract was subjected to silica gel column chromatography eluting with a step gradient of *n-*hexane-ethylacetate (100:0, 90:10, 80:20, 70:30, 60:40, 50:50, 40:60, 30:70, 20:80, 10:90, 0:100, each 200 mL), to obtain 20 fractions (F1-F20) collected on the basis of their TLC profiles. Fractions F10-F14 were recognized as the most interesting ones due to the appearance of white solid, which was washed with hexane and recrystallized from ethanol. This compound was obtained 28.2 mg (0.017%). 

*Compound identification*

^1^H NMR, ^13^C NMR, H-H COSY, HSQC y HMBC spectra (all in CDCl_3_-CD_3_OD) were recorded on Bruker AMX 300 NMR spectrometers, using TMS as internal standard. Mass spectra were recorded on Bruker mass spectrometry using APCI technique positive mode. IR spectra were recorded on a Spectrum RXI FT-IR system (Perkin-Elmer, Waltham, MA, USA) in KBr disks.

*In-vitro biological assays*

*Cell lines and culture medium*

Biological assays were performed using an adenocarcinoma colon cancer cell line (SW480), their derived metastatic SW620 and non-malignant cells (CHO-K1) obtained from The European Collection of Authenticated Cell Cultures (ECACC, England). Cells were cultured in 25-cm^2^ flasks containing Dulbecco’s Modified Eagle Medium, supplemented with 10% heat-inactivated (56 °C) horse serum, 1% penicillin/streptomycin and 1% non-essential amino acids (Gibco Invitrogen, Carlsbad, USA). For all experiments, horse serum was reduced to 3% and the medium was supplemented with 5 mg/mL transferrin, 5 mg/mL selenium and 10 mg/mL insulin (ITS-defined medium; Gibco, Invitrogen, Carlsbad, USA) (11).

*Growth inhibition (SRB)*

The growth inhibition of the ethanol extract, the chromone and 5-fluorouracil (5-FU; the standard drug) was evaluated through sulforhodamine B (SRB) assay, a colorimetric test that is based on staining of total cellular protein of adherent cells. Cells were seeded to a ﬁnal density of 20.000 cells/well in 96-well tissue culture plates and incubated at 37 °C in a humidified atmosphere at 5% CO_2_. All cultures were allowed to grow for 24 h and afterward, they were treated with 1% of DMSO/Ethanol (1:1; vehicle control) or increasing concentrations (0,01 – 0,1 mM) of the compounds. After treatment, cells were fixed with trichloroacetic acid (50% v/v; MERCK) for one hour at 4 °C. Cell proteins were determined by staining with SRB (0.4% w/v; Sigma-Aldrich, United States), then they were washed with 1% acetic acid to remove unbound SRB and left for air-drying. Protein-bound SRB was solubilized in 10 mM Tris-base and the absorbance was measured at 492 nm in a microplate reader (Mindray MR-96A) (11, 12). All experiments were performed in quintuplicate. These values were used to calculate the IC_50_, through dose-response curves for each compound and the selectivity index (SI), by the ratio of IC_50_ values in non-malignant CHO-K1 cells to IC_50_ of SW480 or SW620 cells. SI values higher than 2 were considered selective to cancerous cells over normal cells (13).

*Antiproliferative activity*

The antiproliferative effect of the ethanol extract and the chromone were also tested through sulforhodamine B (SRB) assay. Briefly, cells were seeded to a ﬁnal density of 2500 cells/well in 96-well tissue culture plates and incubated in the same conditions described in the growth inhibition test. The cultures were allowed to grow for 24 h and then, they were treated with increasing concentrations of the ethanol extract of *B. setacea*, the chromone (0,1 – 0,3 mM, ranges depending on the IC_50_ values) or 1% of DMSO/Ethanol (1:1; vehicle control) for 0, 2, 4, 6 and 8 days. Culture media was replaced every 48 h. After each incubation time, cells were fixed, stained, and read as previously described for this technique (11). By the end of the experiment (day 8), cells had an estimated confluence of 90% (SW480) and 70% (SW620).

*Cell Cycle Analysis (Flow Cytometry)*

Cell cycle distribution was analyzed by labeling cells with propidium iodide (PI). Assays were carried out as described by Nicoletti *et al.* (14). In brief, cells were seeded in 6-well tissue culture plates at a density of 2.5×10^5^ cells/well, incubated at 37 °C in a 5% CO_2_ atmosphere. The cultures were allowed to grow for 24 h and then were treated for 48 h with 1% vehicle control (DMSO/ Ethanol 1:1) or the chromone using the mean of the IC_50_ found for SW840 and SW620 cell lines (0.1075 mM). After treatment, cells were collected by scraping and then centrifuged cell pellet was resuspended with phosphate-buffered saline (PBS). The cell suspension was fixed in 1.8 mL 70% ethanol at 4 °C overnight. Afterward, these were centrifuged, washed twice in PBS and resuspended in 300 µL of PBS containing 0.25 mg/mL RNAse (Type I-A, Sigma-Aldrich, Germany) and 0.1 mg/mL PI. Following the incubation in the dark at room temperature for 30 min, the PI ﬂuorescence of 10,000 cells was analyzed using a FACS Canto II ﬂow cytometer and the software BD FACS Diva 6.1.3. (BD Biosciences, San Jose). PI signal was analyzed with excitation at 488 nm, using a Sapphire laser, and fluorescence was detected at 610 nm. Cell clumps were excluded with the PI-Area *vs.* PI-Width signals. The cell cycle model was ﬁxed using the software FlowJo 7.6.2 (Ashland, OR, USA) (14-16).

*Measurement of Mitochondrial Membrane Potential (Δ?m)*

*Ψm)*

Mitochondrial membrane permeability changes were assessed through the fluorescent dye DiOC_6 _(3,3’-dihexyloxacarbocyanine iodide, Thermo Fisher Scientiﬁc, Waltham, MA, USA) and propidium iodide (PI). Cells were seeded to a ﬁnal density of 2.5 × 10^5^ cells/well in 6-well tissue culture plates and allowed to grow for 24 h. Then, these were treated with the chromone using the mean of the IC_50_ found for SW840 and SW620 cell lines (0.1075 mM) or the vehicle control (DMSO/ Ethanol 1%). Later, these were harvested by scrapping at 48 h in the same culture media, stained with DiOC_6 _and propidium iodide (PI) at room temperature for 30 min in darkness. Cells were collected to analyze 10,000 events by ﬂow cytometry with excitation at 488 nm and detection of the emission with the green (530/15 nm) and the red (610/20 nm) ﬁlters. This method allowed us quantifying cells with the depolarized mitochondrial membrane (15, 16).

*Determination of ROS*

SW480 cells were seeded at a final density of 2.5×10^5^ cells/well in 6-well tissue culture plates, allowing them to grow for 24 h. Afterward, these were treated for 48 h with either 1% vehicle control (DMSO/ Ethanol 1:1) or the chromone using the mean of the IC_50_ (0.1075 mM). After treatment, cells were washed with buffer versene and they were collected by trypsinization (0.5% trypsin/2.6 mM ethylenediaminetetraacetic acid) followed by centrifugation. Then, they were resuspended in PBS containing 8μM of CMH2DCFDA, an indicator for the production of reactive oxygen species (ROS) in cells. Subsequently, these were incubated for 30 min, protected from light, at 37 °C, in a humidified atmosphere at 5% CO_2_. Analysis was made by flow cytometry (15). ROS production was considered for the increase in the fluorescence intensity relative to untreated control cells.

*Determination of apoptotic biomarkers*

SW480 and SW620 cells were cultured and treated with chromone or the vehicle control for 48 h. Cells were collected by scraping and lysed with Cell Lysis Buffer (1X, Ref. #9803). The supernatant was used to determine the effect of the chromone on the modulation of apoptotic proteins such as phospho-p53 (Elabscience Biotechnology Co., Ltd., China) and cleaved caspase-3 (Pathscan® (Asp175) Sandwich ELISA Kit, Cell Signaling Technology). Assays were performed according to the manufacturer’s instructions.

*Statistical analysis*

All experiments were performed at least three times. Data are reported as mean ± SE (standard error). Statistical differences between the control group (non-treated) and treated cells were evaluated by one-way ANOVA followed by the Dunnett’s test. Values with *p* ≤ 0.05 were considered significant. Data were analyzed with GraphPad Prism version 8.0.1 for Windows (Graph Pad Software, San Diego, California, USA).

## Results and Discussion

*Chemistry*

*Identification of the chromone*

**Figure 1:**
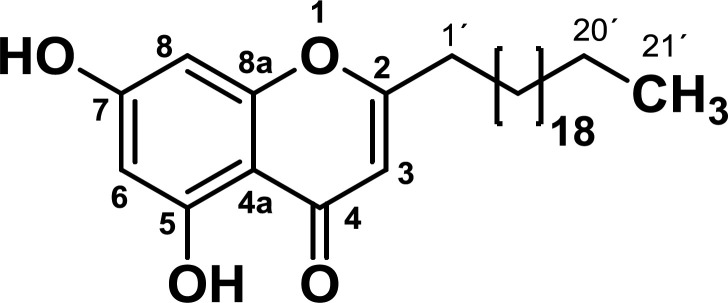
Chemical structure of 2-henicosyl-5,7-dihydroxy-4H-chromen-4-one

The IR spectra showed absorption bands for phenolic, carbonyl, aromatic and aliphatic groups. Analysis of the NMR spectra suggests that the isolated compound has a core of chromone (see spectroscopic data). The mass spectra showed molecular ion [M+H] of 473, corresponding to the molecular weight of 472 g/mol and elemental formula of C_30_H_48_O_4_. The analysis of NMR and MS data led to determine that the structure of the compound corresponded to the 2-heneicosyl-5,7-dihydroxy-4H-chromen-4-one (Figure 1). This compound has already been isolated from the roots of Antidesma *Membranaceum *species (family *Phyllanthaceae*) (17), the stems of *Polygonum aubertii* Henry (18) and the rhizomes of *Dioscorea septemloba *(19), but this is the first time it is reported in the genre *Bomarea*.

2-heneicosyl-5,7-dihydroxy-4H-chromen-4-one: IR (KBr, cm-1): ν max 2917 (C-H), 1657 (C=O), 1633 (C=C), 1588 (C=CAr), 755 (C-HAr). ^1^HNMR (CDCl_3_-CD_3_OD, 300 MHz): δ 0.85 (3H, t, *J* = 6.8 Hz), 1.11-1.46 (36H, m), 1.60-1.76 (2H, m), 2.56 (2H, t, *J* = 7.6 Hz), 5.98 (H3, s), 6.22 (H8, d, *J* = 2.1 Hz), 6.31 (H6, d, *J* = 2.1 Hz), 12.57 (OH). ^13^C NMR (CDCl_3_-CD_3_OD, 75 MHz): δ 14.2 (CH_3_), 23.0 (CH_2_), 27.1 (CH_2_), 29.3 (CH_2_), 29.5 (CH_2_), 29.6 (CH_2_), 29.8 (CH_2_), 30.0 (12 CH_2_), 32.2 (CH_2_), 34.5 (CH_2_), 94.6 (C8), 99.5 (C6), 104.7 (C4a), 107.6 (C3), 158.9 (C8a), 162.0 (C5), 164.6 (C7), 171.3 (C2), 183.1 (C = O). HMBC correlations: 21’ (20´, 19´), 20’ (19´, 18´), 2’ (1´, 2), 1’ (2´, 2, 3), 3 (4, 2, 4a, 1´), 6 (5, 4a, 8), 8 (7, 8a, 6, 4a). MS: m/z 473 (M + H).

*Biological activity*

Chemoprevention, a strategy that is based on the use of natural, synthetic or biological agents which could reverse, suppress or prevent either the initial phases of carcinogenesis or the progression of premalignant cells into invasive disease, has markedly increased the interest in understanding the biology of carcinogenesis in order to identify molecular targets to disturb this process (20, 21). Because of this, the use of different synthetic or natural compounds has emerged as a promising strategy in medicinal chemistry to discover new compounds that could be potential candidates in chemoprevention (22). The aim of the present study was to evaluate the effect of *Bomarea setacea *and the chromone in the modulation of the viability of human adenocarcinoma colon cells with respect to changes in cell cycle, mitochondrial membrane potential, ROS production and some apoptotic proteins since modulation in these processes are key hallmarks of colon cancer (23, 24).

*Growth inhibitory effect of ethanol extract of Bomarea setacea and the chromone on SW480, SW620 and CHO-K1 cells*

To assess the growth inhibitory effect, both the ethanol extract and the chromone were tested *in-vitro* against CHO-K1 cell line, colorectal cancer SW480 cells and its metastatic derivative SW620, through the sulforhodamine B assay. Results were reported as 50% inhibitory concentration (IC_50_ values) and data are summarized in Table 1. According to the results, after 48 h of treatment, the ethanol extract did not exhibit activity against the evaluated cell lines (IC_50_ >10 mM), on the other hand, although the chromone was not as active as the reference drug (chromone: IC_50_/_SW480_ = 0,100 ± 0.006 mM, IC_50/SW620 _= 0,115 ± 0.019 Mm; 5-FU: IC_50_/_SW480_ = 0,042 ± 0,006, IC_50/SW620 _= 0,080 ± 0,027), it displayed better selectivity, being 156-fold more selective against SW480 cells (SI => 100) and 255-fold against SW620 cells (SI => 86,9).

*Antiproliferative effect of Bomarea setacea and the chromone on SW480 and SW620 cells*

**Figure 2:**
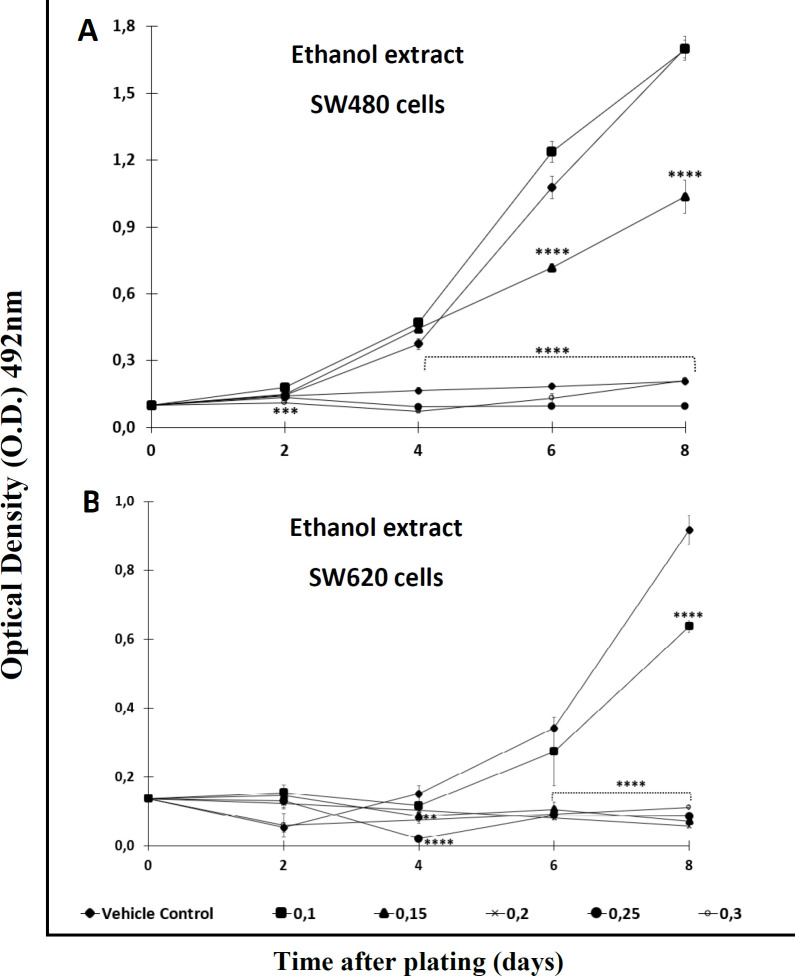
Antiproliferative effect of ethanol extract* of Bomarea setacea* against (A) SW480 cells and (B) its metastatic derivative SW620. Data are presented as the mean ± SE of at least three independent experiments (^**^p < 0.01; ^***^p < 0.001; ^****^p < 0.0001). Optical density (O.D.) is directly proportional to cell mass of adherent cells

**Figure 3:**
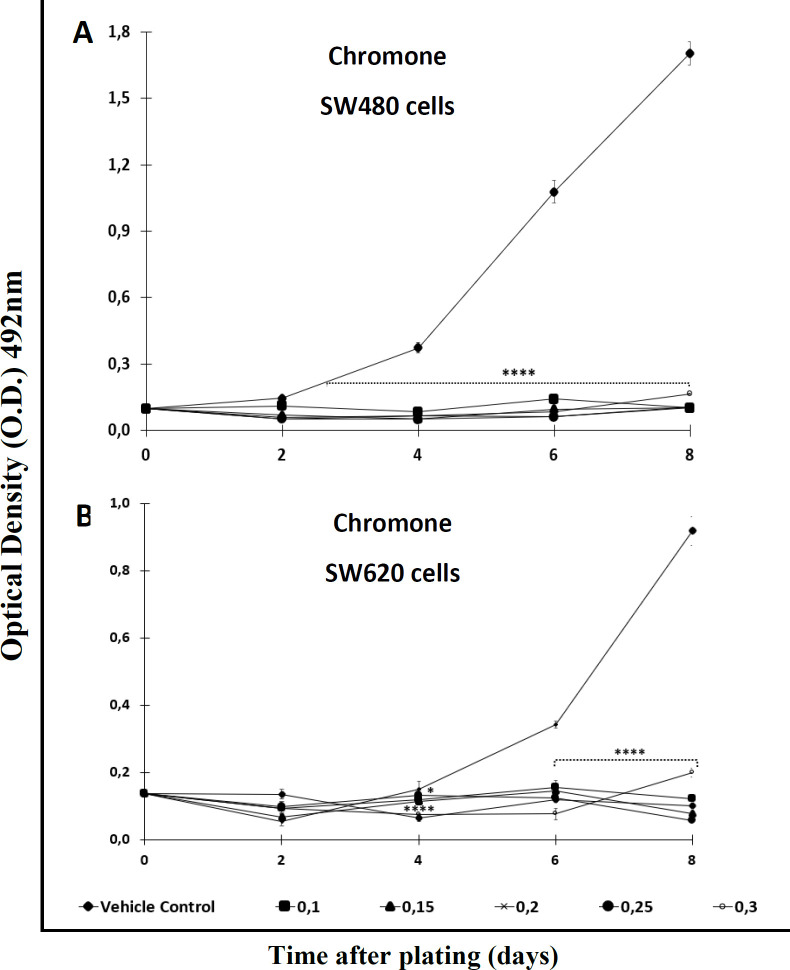
Antiproliferative effect of the chromone from *Bomarea setacea* against (A) SW480 cells and (B) its metastatic derivative SW620. Data are presented as the mean ± SE of at least three independent experiments (^*^p < 0.05; ^****^p < 0.0001). Optical density (O.D.) is directly proportional to cell mass of adherent cells

With the aim of testing the antiproliferative effect of the extract and the Cromone, both compounds were analyzed over a longer period of time. After comparing each treatment with the control, the results indicated that the activity was time- and concentration-dependent. Among the results, it was observed that the ethanol extract required high concentrations (>0,2 mM) to display significant antiproliferative activity against SW480 cells after 2 days of treatment. Besides, this extract required a longer period of time (4 days or upper) to exert effect against the metastatic derivative SW620 (Figures 2A and 2B). On the other hand, the chromone displayed significant antiproliferative activity from day 2 onwards when tested against SW480, even at the lowest concentration evaluated (Figure 3A); however, like the extract, this secondary metabolite required more than 4 days to have significant antiproliferative activity against SW620 cells (Figure 3B) (*p *≤  0.05). In addition, when treated cells were observed with the optical microscope, the cellular morphology was severely perturbed, exhibiting changes in size and shape after treatment. 

*Effect of the chromone on cell cycle distribution of SW80 and SW620 cells*

**Figure 4:**
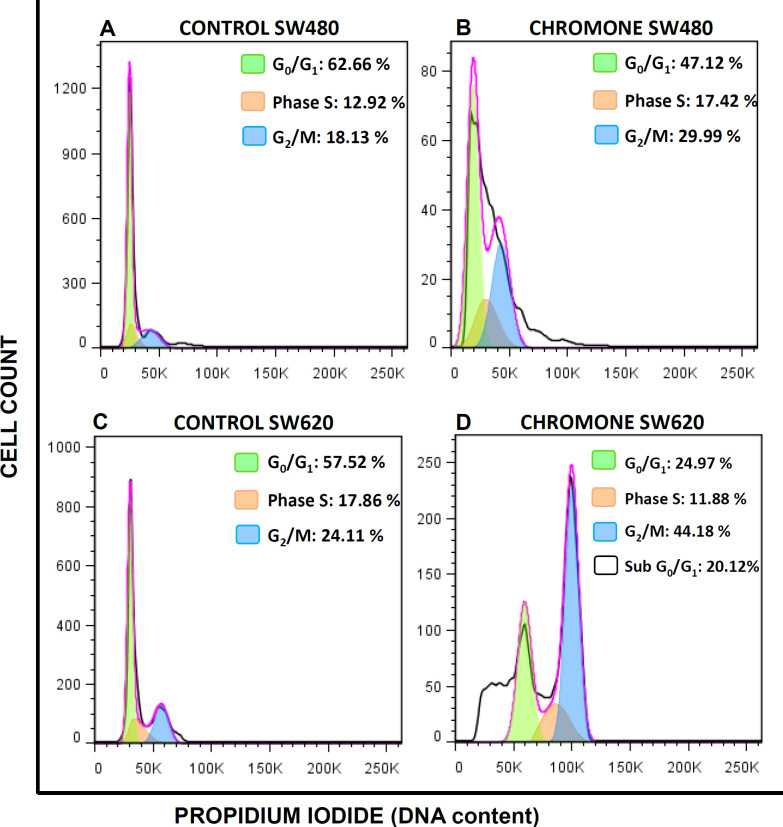
Effect of the chromone on cell cycle distribution of (A-B) SW480 cells (C-D) SW620. One representative image of three independent experiments is shown

**Figure 5:**
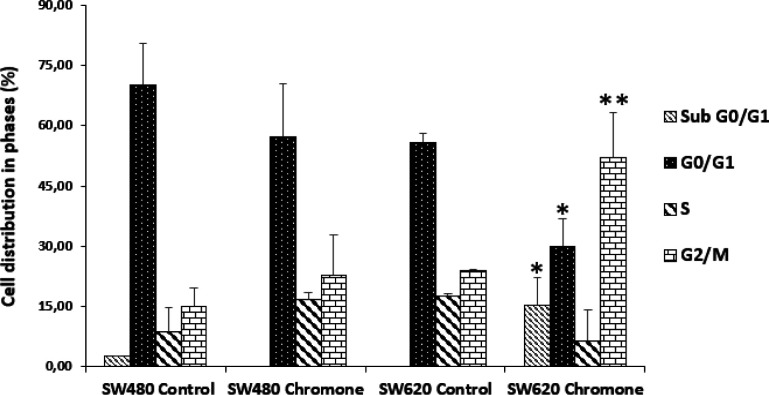
Cell cycle distribution of SW480 and SW620 cells after treatment for 48 h with DMSO/Ethanol (1:1) or the chromone (0.1075 mM). *p*-values lower than 0.05 were considered statistically significant (^*^p < 0.05; ^**^p < 0.01)

The accumulation of mutations in tumor cells contributes to changes in cell cycle distribution, which lead to the proliferation of malignant cells (25). Thus, modulation of this event is seen as a possible target of action in the search for compounds with antitumor activity (26). Because of this, since the chromone exhibited important *in-vitro* growth inhibition and antiproliferative activity, it was further evaluated to determine the effect on the cell cycle distribution of SW480 and SW620 cells. They were treated for 48 h with the mean of the IC_50_. Figure 4 shows the different peaks for each phase of the cycle distribution of SW480 and SW620 cells after treatment. The highest peak represents the cells in G0/G1 phase; the middle region indicates the population in the S phase of the cycle and the final peak on the right represents the cells in the G2/M phase. All results were compared with the control group (SW480: G0/G1 = 62.66%; S = 12.92%; G2/M = 18.13%; SW620: G0/G1 = 57.52%; S = 17.86%; G2/M = 24.11%). According to the results with SW480 cells, the chromone slightly increased the number of cells in the S phase (17.42%) and G2/M (29.99%), with a decrease in G0/G1 (47.12%); however, these changes were not significant (Figure 5).

On the other hand, the results in SW620 cells showed a significant arrest in G2/M (44.18%) with a consequent reduction in G0/G1 (24.97%). Besides, in this metastatic cell line, there was an important population of cells in the subG0/G1 phase, indicating that the chromone induces cell death in this cell line at the conditions evaluated. Similar results were reported by other authors with different chromones. Huang et al. reported in 2009 a series of chromone derivatives with the antiproliferative activity of SW480 and MDA-MB-435s (mammary adenocarcinoma) cells, with dose-dependent effect in G2/M phase after 48 h treatment (27). On the other hand, Mahdavian and colleagues showed that a fusarochromanone produced by a fungus induced antiproliferative effect of SRB12-p9 (malignant skin SCC cells), increasing the fraction of cells in the G2/M phase, probably through an effect on the transition from G2 to mitosis (28). Finally, Tsui and others showed that capillarisin, an active chromone of Artemisia capillaris root extracts, displayed an inhibitory effect of human prostate cancer cells (DU145 and LNCaP) through arrest at the G0/G1 phase (29). All these findings suggest that the chromone moiety could be an important scaffold in anticancer drug discovery.

*Changes in ΔΨm induced by the chromone*

**Figure 6:**
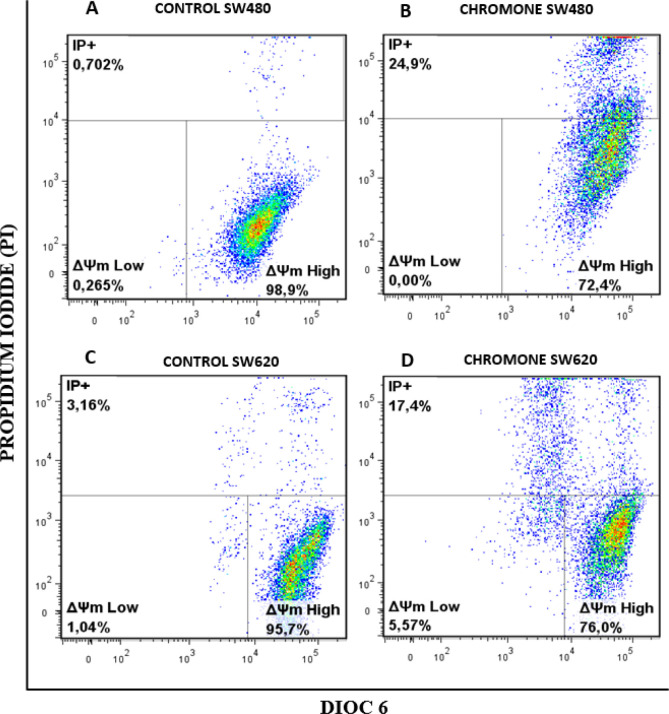
Mitochondrial membrane potential (Δ m) in (A-B) SW480 cells and (C-D) SW620 treated with either the chromone or 1% DMSO/Ethanol (1:1) as control, during 48 h. Flow cytometric analysis of cells stained with DiOC6 and PI; Δ m High: live cells with high membrane polarization; Δ m Low and PI+: cells in latency that lose membrane polarization and dead cells, respectively

Since mitochondria play key roles in activating apoptosis (30-32), and due to the previous results of viability and antiproliferative activity of the chromone, we evaluated the ability of this metabolite to induce changes in the ΔΨm that could cause mitochondrial dysfunction of SW480 and SW620 malignant cell lines. After 48 h of treatment with the chromone or DMSO/Ethanol 1% (1:1; vehicle control), SW480 and SW620 cells were stained with the carbocyanine dye DiOC6 and propidium iodide (PI) and analyzed by flow cytometry. DiOC6, a fluorescent dye that accumulates in mitochondria due to its large negative membrane potential, is released to the cytosol after a membrane depolarization (membrane with reduced ΔΨm), staining intracellular membranes (15, 16). Figure 6 shows the results for the ΔΨm. The right lower quadrant (ΔΨm High) indicates the amount of live cells with high membrane polarization. The left lower region (ΔΨm Low) and the upper quadrant (PI+) indicate cells in latency that lose membrane polarization, with membrane damage and dead cells. 

**Figure 7:**
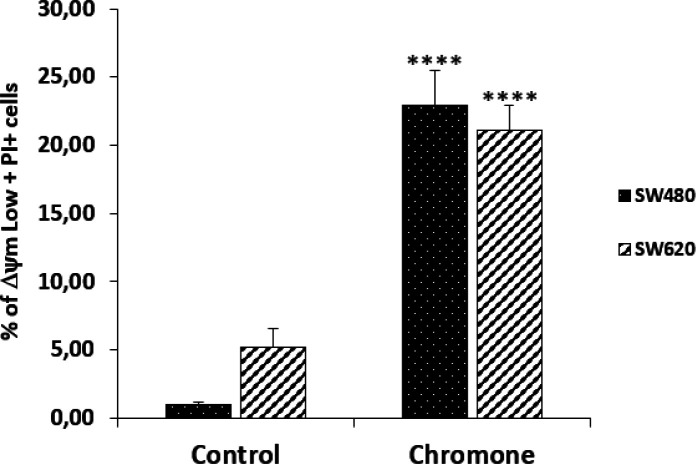
Representation of data with total Δ m Low plus PI+ cells in bar chart form. Data are presented as the mean of these two populations ± SE of three independent experiments. *p*-values lower than 0.05 were considered statistically significant (^****^p < 0.0001).

According to the results, the chromone caused a significant loss in mitochondrial membrane potential, as shown in the population with ΔΨm High (SW480 = 72.4%; SW620 = 76%) regarding the control group (SW480 = 98.9%; SW620 = 95.7%). Besides, this chromone significantly increased the population with positive staining for PI in SW480 and SW620 cells (24.9% and 17.4%, respectively). Cells in latency together with dead cells are clearly seen in Figure 7 with their statistical significance. All these results suggest that the chromone evaluated induce damage in cell membrane and death in SW480 and SW620 cells at the conditions tested. 

*Determination of ROS*

The formation of radical oxygen species (ROS) is a normal process in the body, regulated by different antioxidant systems. This is an equilibrated activity for maintaining low doses of these molecules to preserve their physiological functions since they regulate a variety of cellular signaling pathways, among them, proliferation, migration, cell cycle progression, differentiation, and cell death. When ROS quantity dramatically increases due to an imbalance between production and elimination, there is an association with oxidative stress and signal transduction, which causes damage to membranes, lipids, nucleic acids, proteins and organelles, leading to activation of cell death processes, such as apoptosis (33-35). Because of this, since the chromone induced important damage to cells in the previous mitochondrial membrane potential test, we evaluated it in order to determine if it induces oxidative stress that could be associated with this mitochondrial damage. We used the indicator CM-H2DCFDA to know the effect on the production of radical oxygen species (ROS). It is a non-fluorescent dye that passively diffuses into cells, where the acetate groups are cleaved by intracellular esterases. Subsequent production of ROS induces oxidation, producing a fluorescent compound that is trapped inside the cell. After measured the fluorescence intensity of CM-H2DCFDA through flow cytometry, we did not observe changes in ROS production (data not shown) after treatment with the chromone, suggesting this molecule does not act as an antioxidant or prooxidant agent. Similar findings were reported by Yasser *et al.* (36), who evaluated the antioxidant effect of a different chromone using a cellular lipid peroxidation antioxidant activity (CLPAA) in HepG2 cells.

*Effect of the chromone on the expression of apoptotic biomarkers*

**Figure 8:**
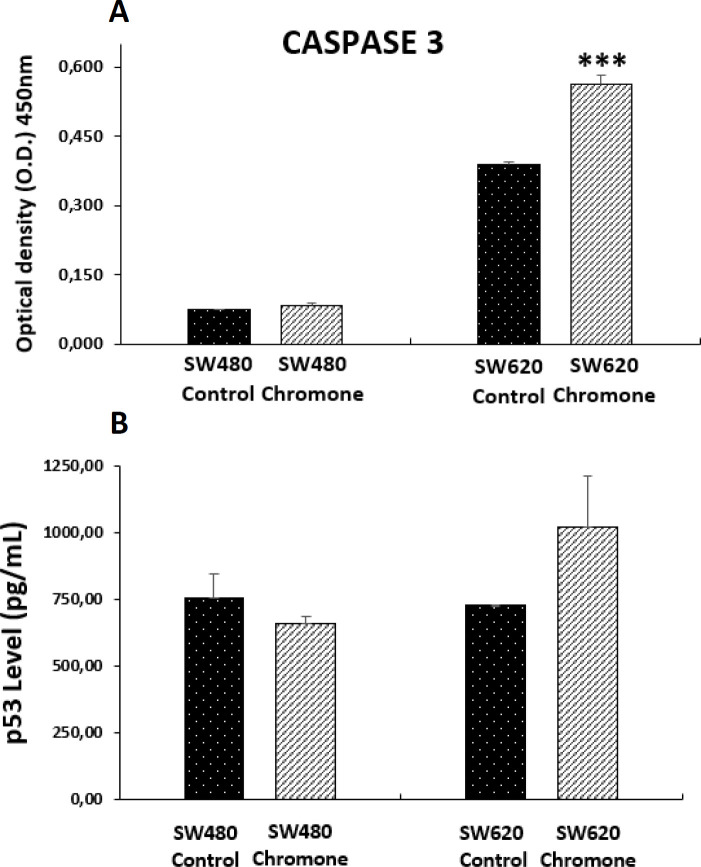
Level of apoptotic biomarkers in SW480 and SW620 cells 48 h post-treatment with 0.1075 mM of the chromone or 1% DMSO/Ethanol (Control). (A) Levels of cleaved caspase 3. (B) Levels of the active form of tumor suppressor protein p53. Data are represented as the average of two independent experiments. The optical density (O.D.) is directly proportional to the amount of protein of living cells. *p*-values lower than 0.05 were considered statistically significant (^***^p < 0.001).

Deficient apoptosis is often associated with numerous human cancers; thus, regulation of apoptotic cell death by targeting antiapoptotic and proapoptotic proteins is an important strategy for anticancer drug discovery that is needed to find more selective agents able to increase efficacy and reduce side effects. The apoptotic pathway involves the sequential activation of caspases, a family of proteases classified as an initiator (caspase-8 and -9) or executioner caspases (caspase-3, -6, and -7). Among them, caspase 3 is considered one of the most important and its activation is a critical step in the pathway leading to cell shrinkage, membrane blebbing and DNA fragmentation, biochemical and morphological changes that underlie apoptosis (37). Because of this, we tested the effect of the chromone on the levels of the active form of this protease in colon adenocarcinoma cells after 48 h of treatment. We observed that this compound induced a significant increase of cleaved caspase 3 in SW620 cells, suggesting a possible mechanism of apoptosis in an *in-vitro* model of CRC (Figure 8a). Similar results were reported by Mahdavian and colleagues, which demonstrated that another chromone isolated from a fungus (fusarochromanone) induced the cleavage of caspase 3 in MDA-MB-231 breast cancer cells, initiating degradation in the final stages of apoptosis (28). In addition, given the importance of the tumor-suppressor protein p53 in regulating different cellular processes such as apoptosis (38), the effect of the chromone on this protein was also tested. Although this protein is mutated in SW480 and SW620 cells, which results in an abnormal protein, other authors have demonstrated that it is possible to activate it both *in-vitro* and *in-vivo* through different mechanisms (39, 40), suggesting that p53 retains some of the functions as it maintains residual DNA-binding ability (39). Because of this, we evaluated this protein in the mentioned colon cancer cells. According to the results, we found that the chromone did not induce the increase in the active form of p53 (Figure 8b), suggesting a different pathway in the apoptotic process, independent from p53.

**Table 1:** *In-vitro* cytotoxic activity and selectivity index of ethanol extract* of Bomarea setacea, *the chromone and the reference drug (5-FU).

**48 (h)**						
Cell line	**Ethanol extract**		**Chromone**		**5-fluorouracil**	
	**IC**_50 _ **(mM)**	**SI**	**IC**_50 _ **(mM)**	**SI**	**IC**_50 _ **(mM)**	**SI**
CHO-K1	>10	----	>10	----	0,027 ± 0,002	----
SW480	>10	>1	0,100 ± 0,006	>100^****^	0,042 ± 0,006	0,64
SW620	>10	>1	0,115 ± 0,019	>86,9^****^	0,080 ± 0.027	0,34

The IC_50_ values were obtained from dose-response curves for each compound. Selectivity index (SI) was calculated by the ratio of IC_50_ values in non-malignant CHO-K1 cells to IC_50_ of SW480 or SW620 cells. Data are presented as the mean ± SE of at least three independent experiments. *p*-values lower than 0.05 were considered statistically significant (^****^p < 0.0001).

## Conclusion

We showed for the first time that the 2-henicosyl-5,7-dihydroxy-4H-chromen-4-one (termed the chromone through the text) isolated from *Bomarea setacea*, induces death in two different adenocarcinoma cells, involving selective growth inhibition, antiproliferative and proapoptotic effect, evidenced by the increase of caspase 3, with an important arrest in G2/M and the presence of cells in subG0/G1 phase in the metastatic derivative SW620. Besides, the loss of mitochondrial membrane potential with damage in the cell membrane that was observed in both cell lines (SW480 and SW620), together with the changes in size and shape after treatment, suggest the chromone induces this process of death in an *in-vitro* model of CRC. In none of these cases were observed changes in p53 activation or ROS production, suggesting that the process of death is independent of these two events. Our findings suggest that this compound could be a promising chemopreventive agent against colorectal cancer and thus, it is necessary to carry out further studies.
